# Antiviral resistance in HCV strains isolated from Romanian patients with limited treatment options for chronic HCV infection

**DOI:** 10.1186/1471-2334-14-S7-P8

**Published:** 2014-10-15

**Authors:** Anca Streinu-Cercel, Oana Săndulescu, Daniela Manolache, Dragoş Florea, Dan Oțelea, Adrian Streinu-Cercel

**Affiliations:** 1 Carol Davila University of Medicine and Pharmacy, Bucharest, Romania; 2 National Institute for Infectious Diseases "Prof. Dr. Matei Balş", Bucharest, Romania

## Background

With the advent of direct-acting antivirals, the rate of sustained virologic response (SVR) after treatment for chronic HCV infection has dramatically increased [1]. However, recent data show that there is cause for concern regarding the potential emergence of viral resistance [2].

## Methods

We performed a study to assess the antiviral resistance profile in a series of patients with limited treatment options for chronic HCV infection.

## Results

We assessed data from 12 patients (gender ratio 1:1), of which 10 had HCV monoinfection and 2 had HCV+HIV coinfection. The mean age was 43.8±16.5 years (range 19-71 years). Eleven patients were infected with HCV genotype 1b, and one patient had been coinfected with genotype 2a/2c but had spontaneously cleared 2a infection and was now monoinfected with HCV 2c.

Only one patient had IL28-B genotype CC, 4 patients CT and 5 patients TT (in two cases data on IL28-B were not available). Seven of the patients had received prior anti-HCV therapy: 2 with peg-interferon+ribavirin, 2 with faldaprevir-based regimens and 3 with telaprevir-based regimens. Of them, 3 had been non-responders and 4 had been relapsers.

The mean plasma HCV-RNA was 6.1±0.7 log_10_ IU/mL. Patients were distributed over the whole range of fibrosis values on FibroMax, with a slight predominance of advanced fibrosis: F3 (2 patients) and F4 (3 patients).

Resistance to boceprevir or simeprevir was identified in 3/12 cases (fold-change range: 4-24) and 2/12 cases (fold-change range: 32-38), respectively, although none of the patients had received prior therapy with these antivirals.

Resistance to telaprevir was identified in 3/12 cases (surprisingly, none of the cases with telaprevir therapy). Possible resistance was identified in another 6 cases (including cases treated with telaprevir and faldaprevir). The overall fold-change range was 1.8-22.4.

Resistance to faldaprevir was identified in 3/12 cases (surprisingly, none of the cases with faldaprevir therapy), with a fold-change range of 1.2-360.0.

## Conclusion

Cross-resistance to HCV protease inhibitors (PI) remains a cause for concern, particularly in patients with history of treatment with HCV PI-based regimens.
